# The Modes of Dysregulation of the Proto-Oncogene T-Cell Leukemia/Lymphoma 1A

**DOI:** 10.3390/cancers13215455

**Published:** 2021-10-29

**Authors:** Johanna Stachelscheid, Qu Jiang, Marco Herling

**Affiliations:** 1 Department I of Internal Medicine, Center for Integrated Oncology (CIO), Aachen-Bonn-Cologne-Duesseldorf, Excellence Cluster for Cellular Stress Response and Aging-Associated Diseases (CECAD), Center for Molecular Medicine Cologne (CMMC), University of Cologne, 50931 Cologne, Germany; johanna.stachelscheid@uk-koeln.de (J.S.); qu.jiang@uk-koeln.de (Q.J.); 2 Department of Hematology, Cellular Therapy, and Hemostaseology, University of Leipzig, 04103 Leipzig, Germany

**Keywords:** TCL1 oncogenes, kinase signaling, lymphoma, T-PLL, CLL, BPDCN

## Abstract

**Simple Summary:**

T-cell leukemia/lymphoma 1A (*TCL1A*) is a proto-oncogene that is mainly expressed in embryonic and fetal tissues, as well as in some lymphatic cells. It is frequently overexpressed in a variety of T- and B-cell lymphomas and in some solid tumors. In chronic lymphocytic leukemia and in T-prolymphocytic leukemia, TCL1A has been implicated in the pathogenesis of these conditions, and high-level TCL1A expression correlates with more aggressive disease characteristics and poorer patient survival. Despite the modes of TCL1A (dys)regulation still being incompletely understood, there are recent advances in understanding its (post)transcriptional regulation. This review summarizes the current concepts of TCL1A’s multi-faceted modes of regulation. Understanding how TCL1A is deregulated and how this can lead to tumor initiation and sustenance can help in future approaches to interfere in its oncogenic actions.

**Abstract:**

Incomplete biological concepts in lymphoid neoplasms still dictate to a large extent the limited availability of efficient targeted treatments, which entertains the mostly unsatisfactory clinical outcomes. Aberrant expression of the embryonal and lymphatic TCL1 family of oncogenes, i.e., the paradigmatic *TCL1A*, but also *TML1* or *MTCP1*, is causally implicated in T- and B-lymphocyte transformation. TCL1A also carries prognostic information in these particular T-cell and B-cell tumors. More recently, the *TCL1A* oncogene has been observed also in epithelial tumors as part of oncofetal stemness signatures. Although the concepts on the modes of TCL1A dysregulation in lymphatic neoplasms and solid tumors are still incomplete, there are recent advances in defining the mechanisms of its (de)regulation. This review presents a comprehensive overview of TCL1A expression in tumors and the current understanding of its (dys)regulation via genomic aberrations, epigenetic modifications, or deregulation of TCL1A-targeting micro RNAs. We also summarize triggers that act through such transcriptional and translational regulation, i.e., altered signals by the tumor microenvironment. A refined mechanistic understanding of these modes of dysregulations together with improved concepts of TCL1A-associated malignant transformation can benefit future approaches to specifically interfere in TCL1A-initiated or -driven tumorigenesis.

## 1. Introduction

T-cell leukemia/lymphoma 1A (*TCL1A*) was first described as a proto-oncogene in hematological neoplasms between 1989 and 1994 [1,2,3]. It is the prototype of a 3-paralogue gene family, further including *TCL1B* and mature T-cell proliferation 1 (*MTCP1*) [4]. Their small proteins share high sequence homology [1,5,6] and consist of a common three-dimensional structure of an orthogonal 8-stranded β-barrel with a hydrophobic core and a unique topology [7].

Physiologically, the expression of TCL1A is restricted to embryonic tissues and to pre-mature B cells and T cells, suggesting its role in reproduction and adaptive immunity, which could be corroborated in sub-total knockout mice [8,9]. Its aberrant overexpression was first identified in T-cell prolymphocytic leukemia (T-PLL) via genomic aberrations involving its locus at chromosome 14 [1]. In contrast, in B-cell tumors, there is a virtual absence of such rearrangements [10] or gain-of-function mutations [11] involving the *TCL1A* locus. In these tumors, TCL1A expression parallels its regulation in non-neoplastic B cells [12].

The T- and B-cell oncogenic potential of human TCL1A was formally shown in transgenic (tg) mice [13,14,15,16]. When ectopically expressed in T cells under the proximal *Lck^pr^*-promoter (*Lck-TCL1A*) or in B cells under the *VH*-promoter/*IgHµ* enhancer (*Eµ-TCL1A*), the mice develop a disease closely resembling human T-PLL or chronic lymphocytic leukemia (CLL), respectively. A related model, *pEμ-B29-TCL1A* mice, produces germinal center (GC)-derived B-cell tumors that resemble Burkitt lymphoma (BL), follicular lymphoma (FL), and diffuse large B-cell lymphoma (DLBCL), and in one founder line also a T-PLL-like disease [15]. TCL1A tg mice, in particular the well-established *Eµ-TCL1A* model for CLL, have been crossed with a variety of other alleles (reviewed in [17,18]). This enabled the investigation of novel pathogenic mechanisms, such as interactions with the microenvironment (e.g., *Eµ-TCL1A*; *CD44^−/−^* [19]; *Eµ-TCL1A*; *CXCR4^C1013G^* [20]) or signaling (e.g., *Eµ-TCL1A*; *pkcβ^−/−^* [21]; *Eµ-TCL1A^tg/wt^*; *Cd19^Cre/wt^*; *R26-fl-Akt-C* [22]), as well as the role of recurrent genomic lesions (e.g., *Eµ-TCL1A*; *CD19^cre/wt^*; *Trp53^fl/fl^* or *Eµ-TCL1A*; *CD19^cre/wt^*; *Atm^fl/fl^* [23]).

In T-PLL and CLL patients, the expression of tumor-associated TCL1A mRNA and protein shows considerable inter-patient variability. Notably, high levels of TCL1A correlate with aggressive clinical features (e.g., leukemic burden, growth kinetics), high-risk cytogenetics, poorer responses to chemo-immunotherapies, and inferior clinical outcomes [24,25,26,27,28,29]. Therefore, TCL1A has been established as a prognostic marker in both entities. Additionally, in solid tumors, the first evidence links high TCL1A expression to adverse clinical features and outcomes [30].

Given TCL1A’s important role in tumor initiation, progression, and maintenance, investigating the modes of its oncogenic function and dysregulation can help to better understand the pathogenesis of these neoplasms and to contribute to the identification of possible new treatment targets. This review summarizes the current knowledge on the spectrum of TCL1A’s modes of upstream regulations in normal and transformed lymphocytes, as well as in stem cells and solid tumors.

## 2. The Normal and Tumor-Associated Expression of TCL1A

### 2.1. The Physiological Expression of TCL1A

The expression of TCL1A is normally restricted to embryonic tissues and pre-mature B cells and T cells and is conserved to some mammals [31]. Its expression in embryonic tissues could be observed in murine early cleavage embryos, where it shuttles between the cortex and the nucleus during the first cleavages until it declines during transition through the blastocyst stage [9]. In humans, its expression could be detected (rather in cell subpopulations) in fetal liver, kidney, and thymus, while in adult organisms, only testes, spleen, tonsil, colon, and bone marrow harbor TCL1A-positive cells (with hematopoietic stem cells likely being negative) [1,5,30]. Plasmacytoid dendritic cells (pDCs) [32] and lymphocyte subsets express TCL1A [1].

T cells lose TCL1A expression starting at the CD4/CD8 double-positive (DP) thymocyte stage, and a role for TCL1A in pre-TCR signaling appears attractive to speculate; post-thymic T cells with matured TCRs no longer express TCL1A [1,33]. It is believed that during these physiological *TRA* locus rearrangements, which exchange the pre-TCR to the TCR at that DP stage, erroneous reassemblies of *TRA* regions juxtapose the *TCL1A* locus under control of regulatory elements of *TRA/D* genes, causing aberrant TCL1A expression toward T-PLL [34].

In B cells, TCL1A experiences a drastic downregulation starting at the entry of the highly TCL1A-positive mantle-zone B cell into the GC environment. TCL1A is completely silenced in terminally differentiated B cells, such as plasma cells [12,35].

### 2.2. Expression of TCL1A in Hematologic Malignancies

Overexpression of TCL1A in the neoplastic context was first identified in T-PLL, where translocations or inversions juxtapose the gene locus at 14q32 to highly active regulatory elements of T-cell receptor (TCR) genes [1]. This constitutive expression counters the physiological silencing of TCL1A in maturing T cells. Interestingly, a germline duplication of the chromosomal locus 14q32 including the *TCL1A* gene was identified in several families with an autosomal dominant myeloid neoplasm predisposition syndrome. However, TCL1A’s role in its pathogenesis is still under investigation [36,37].

In B-cell tumors, TCL1A expression mostly parallels its regulation in non-neoplastic B cells. Those of pre-GC derivation, such as B-cell acute lymphoblastic leukemia/lymphoma (ALL/LBL) and mantle cell lymphoma (MCL), are highly TCL1A positive [12]. Hodgkin lymphoma (HL), and post-GC tumors such as splenic- and mucosa-associated lymphoid tissue (MALT) types of marginal zone lymphoma (MZL), as well as multiple myeloma (MM), are all consistently negative for TCL1A [12]. In CLL, the immunoglobulin heavy chain variable (*IGHV*) region gene unmutated subtype of pre-GC origin shows higher TCL1A levels than the *IGHV*-mutated TCL1A^low^ CLL subset [24]. BL shows rather a uniform expression of TCL1A [12,38]. FL shows variable TCL1A levels with a loss of expression in higher-grade tumors, while TCL1A expression is found less frequently in DLBCL, particularly in the subset of the activated B-cell type [12].

### 2.3. Expression of TCL1A in Solid Tumors

Recent years have seen an increasing number of publications that also implicate TCL1A in stemness programs of non-hematopoietic cancers. It was unexpected to find TCL1A to be expressed in epithelial solid tumors such as breast or colorectal cancers (CRC) [30]. Interestingly, such oncofetal patterns of TCL1A expressions already had been implicated by its detection in a high proportion of testicular seminomas, ovarian dysgerminomas, and in unclassified intratubular germ cell neoplasms [9,39,40,41]. Fittingly, TCL1A is part of molecular stem-cell marker signatures (including OCT3/4, NANOG, SOX2, etc.) that are detected in carcinomas of the bladder, prostate, colon, and liver [42,43,44]. It was also implicated that TCL1A, together with consensus transcriptional regulators of tumor stem cells (e.g., OCT3/4, SOX2), promotes the transformation of Barrett’s esophagus to adenocarcinoma, with an increased expression in Barret’s over normal (negative) esophageal mucosa [45]. Table 1 provides a summary of the malignancies that express TCL1A.

### 2.4. Clinical Impact of Detection of TCL1A

Given the apparently histogenetically fixed expression of TCL1A in lymphoid tumors, the expression of TCL1A harbors important diagnostic information. Due to its specific expression in T-PLL among other mature T-cell lymphomas (MTCL) with prominent peripheral blood (PB) presentation, TCL1A was established as a first-order marker of high specificity [46,55]. It is now included in a widely accepted algorithm to differentiate WHO-recognized subsets of leukemic T-cell tumors, which markedly differ in their treatment and prognosis. Hard-to-classify cases of leukemic MTCL, especially those showing similar clinical features, e.g., skin lesions of T-PLL vs. those of primary cutaneous T-cell lymphomas (CTCL) are now nearly unequivocally assigned by means of TCL1A expression [56,57,58].

Importantly, detection of TCL1A expression in the former category of CD4^+^ CD56^+^ blastic tumors of skin (previously thought to be of NK origin), helped to reclassify them as a blastic plasmacytoid dendritic cell neoplasm (BPDCN) of (pre)-pDC origin. TCL1A is now a core marker in its differential diagnosis, which has drastic prognostic and therapeutic implications [32]. As the expression of TCL1A in B-cell tumors mainly parallels regulation in non-neoplastic B cells, its expression can be used to distinguish B-cell tumors of pre-GC origin from those of post-GC origin [12].

In addition to diagnostic value, TCL1A mRNA and protein expression carry prognostic information in several leukemias/lymphomas. In CLL and T-PLL, higher TCL1A levels correlate with more aggressive disease features, such as higher white blood cell (WBC) counts and faster tumor cell doubling, as well as a shorter overall/progression-free survival [24,26,27]. Furthermore, high TCL1A levels correlate with a more pronounced T-cell or B-cell receptor responsiveness and by that functionally define subsets of T-PLL [26] and CLL [24], respectively, which may guide future inhibitory designs for more individualized treatments.

Across non-Hodgkin lymphomas, a gene set enrichment analysis associated high TCL1A levels with important pathways controlling B-cell lymphomagenesis, including, e.g., B-cell receptor, NF-κB signaling, cell death, and MAP kinase, implicating a central role of elevated TCL1A expression in their pathogenesis and aggressiveness [38]. In line with this, high TCL1A was correlated with shorter leukemia-specific survival in MCL [38], as well as with clinical stage and shorter overall survival in DLBCL [50].

Additionally, in some solid tumors, TCL1A can be utilized as a prognostic marker. In CRC, high TCL1A correlates with tumor differentiation and clinical stage and is an independent factor for CRC-specific and disease-free survival. Furthermore, it predicts the outcome of stage II/III patients who receive standard adjuvant chemotherapy [30]. In hepatocellular carcinoma (HCC), high TCL1A levels in patients under sorafenib treatment correlate with an inferior overall and progression-free survival [44].

## 3. The Physiological and Disease-Associated Function of TCL1A

**Figure 1:**
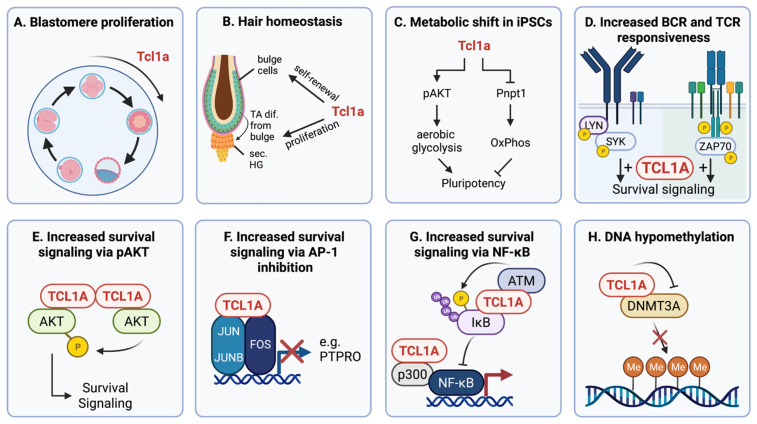
TCL1A functions as a pleiotropic adapter molecule in stemness and survival signaling. Note that “roles” and “modes of actions” at various levels (e.g., cell function, impacted pathway, concise molecular interaction) are highlighted and are in part artificially separated (i.e., E part of C and D): (**A**) in murine blastomeres, Tcl1a is important in early proliferation, as Tcl1a^−/−^ mice show a block of blastomere development at the 8-cell stage [9]; (**B**) Tcl1a regulated hair growth, as shown by hair loss in Tcl1a^−/−^ mice. Tcl1a is expressed in the bulge cells (stem cell niche) and in the secondary hair germ/transit-amplifying (TA) cells (proliferative structure) during the catagen–telogen (resting phase) transition and early anagen stage (regeneration phase). In Tcl1a^−/−^ mice, the bulge cells show reduced expression of the stem cell marker CD34. Furthermore, a Tcl1a knockout led to reduced proliferation of TA cells, needed for new hair formation [59]; (**C**) upregulation of Tcl1a leads to metabolic shifts toward aerobic glycolysis via activation of Akt and repression of Pnpt1, thereby contributing to pluripotency of induced pluripotent stem cells (iPSCs) [60]; (**D**) in cells of chronic lymphocytic leukemia (CLL) and T-cell prolymphocytic leukemia (T-PLL), TCL1A increases the responsiveness to B-cell receptor (BCR) and T-cell receptor (TCR) stimulation, respectively, by a kinase activating effect [24,26,61]; (**E**) interaction of the TCL1A homodimer with AKT molecules leads to augmented trans-phosphorylation and catalytic activity of the oncogenic Ser/Thr kinase AKT, resulting in increased survival signaling [62]; (**F**) the interaction of TCL1A with AP-1 components—namely, JUN, JUNB, and FOS, leads to impaired AP-1 signaling and thereby sustained anti-apoptotic signals [11]; (**G**) TCL1A interacts with IκB and mediates its phosphorylation via ATM, leading to its subsequent ubiquitination-dependent degradation. Inhibition of this negative regulator IκB causes increased NF-κB signaling, which is additionally strengthened by the TCL1A-p300 interaction [11]; (**H**) physical interaction of TCL1A with DNMT3A reduces the methyltransferase activity of DNMT3A, which leads to a higher number of hypomethylated genomic regions [63], which is implicated in the pathogenesis of CLL [64]. This figure was created using BioRender.com (accessed on 22 October 2021).

The expression of TCL1A in embryonic tissues, as well as its recurrent overexpression and prognostic/predictive value in different malignancies, implicates an important function of TCL1A in key signaling pathways mediating stemness and survival (Figure 1).

### 3.1. The Functional Role of TCL1A in Embryonic Development and Stemness

Generally, the 14 kDa TCL1A protein lacks kinase activity and a DNA-binding motif. Instead, its eight-stranded β-barrel with a hydrophobic core suggests its binding to small hydrophobic ligands [7]. Its currently best-established function is enhancing the catalytic activation and mediating the nuclear translocalization of the oncogenic Ser/Thr kinase Akt by interacting directly with its pleckstrin homology domain [62,65,66,67,68].

There is increasing evidence for the role of TCL1A in embryonic development. The reduced fertility observed in *Tcl1a^−/−^* female mice was ascribed to a frequent block of blastomere proliferation beyond the eight-cell stage, despite displaying normal major differentiative traits [9]. Furthermore, these mice show defects in hair formation and skin homeostasis, which can be attributed to the role of Tcl1a in maintaining the self-renewal, proliferation, and apoptosis of bulge cells and keratinocytes [59,69]. An effect of Tcl1a on proliferation, but not differentiation, was also validated in murine embryonic stem cells (mESCs) and could be at least in part explained by an increase in Akt activation [70,71]. In contrast, others identified Tcl1a as a member of an interconnected transcriptional network regulating self-renewal of mESCs in vitro by blocking the differentiation into epiblast-derived lineages [72]. Furthermore, Tcl1a is involved in the reprogramming of murine-induced pluripotent stem cells (iPSCs). It is expressed late in the reprogramming process and partly regulates the metabolic shift from oxidative phosphorylation to glycolysis. This is mediated by activating Akt to activate glycolysis and by inhibiting the mitochondrial polyribonucleotide nucleotidyltransferase 1 (PNPT1, also called PNPase) to diminish oxidative phosphorylation [60]. Fittingly, PNPT1 was shown to interact with TCL1A in B cells, however, with yet unresolved functional consequences [73]. In contrast to the findings in iPSCs, the introduction of TCL1A into mature B-cell lymphoma lines led to reduced aerobic glycolysis and a higher rate of oxygen consumption coupled to ATP-synthesis [74].

### 3.2. The Functional Role of TCL1A in Cancer Signaling and Pathogenesis

The adverse clinical outcomes in some solid tumors and leukemias in association with high TCL1A expression are likely to a considerable part the result of the TCL1A-mediated augmented activity of AKT contributing to enhanced proliferation and multi-nodal resistance [26,35,75,76]. However, the sole activation of AKT was unable to recapitulate the oncogenic function of TCL1A overexpression, suggesting a more complex functional spectrum of this unconventional oncogene [77,78]. Indeed, in recent years, more pathways that are modulated by TCL1A have been identified. Via interacting with ataxia-telangiectasia-mutated (ATM) [79] and p300/cAMP response element-binding protein (CREB) [11], TCL1A has been linked to contributing to accelerated tumorigenic NF-κB signaling, as important in CLL pathogenesis. By determining the magnitude and quality of TCR and B-cell receptor (BCR) responses in T-PLL and CLL, respectively, mostly through a kinase-enhancing effect [24,26], TCL1A provides survival advantages through threshold-lowering effects in the context of dependence on low-level (tonic) antigen receptor input. Inhibition of activator protein 1 (AP-1) transcriptional activity via interaction of TCL1A with the AP-1 complex represents another mechanism to antagonize expression of pro-apoptotic factors, such as the protein tyrosine phosphatase receptor type O (*PTPRO*) [11,80].

There is also evidence for TCL1A to form a functional synergism with hypomorphic ATM toward a prominent phenotype of deficient DNA damage responses in T-PLL [27]. Overexpressed TCL1A promoted increased reactive oxygen species levels, telomere attrition, alongside impaired sensing and protracted processing of DNA double-strand breaks upon genotoxic stress [27].

TCL1A was also shown to contribute to epigenetic reprogramming via interacting with the de novo DNA methyltransferase 3A (DNMT3A) and reducing its enzymatic activity. Accordingly, B cells from *Eµ-TCL1A* mice show more hypomethylated regions than age-matched wild-type cells [63]. In several leukemia mouse models, Dnmt3a has been identified as a tumor suppressor [64], therefore suggesting a meaningful impact of TCL1A-mediated inhibition of DNMT3A during leukemogenesis.

In HCC, TCL1A overexpression is implicated in mediating metabolic alterations. TCL1A enhances the pre-mRNA splicing and thereby protein expression of the glucose-6-phosphate dehydrogenase (G6PD) by interacting with the heterogeneous nuclear ribonucleoprotein (hnRNPK), leading to an increased pentose phosphate pathway flux and glucose consumption [44].

In summary, based on the heterotypic functions of TCL1A on different signaling branches, its transforming impact is most likely a synergistic net effect of several dysregulated pathways.

## 4. The Modes of TCL1A (Dys)Regulation

**Figure 2:**
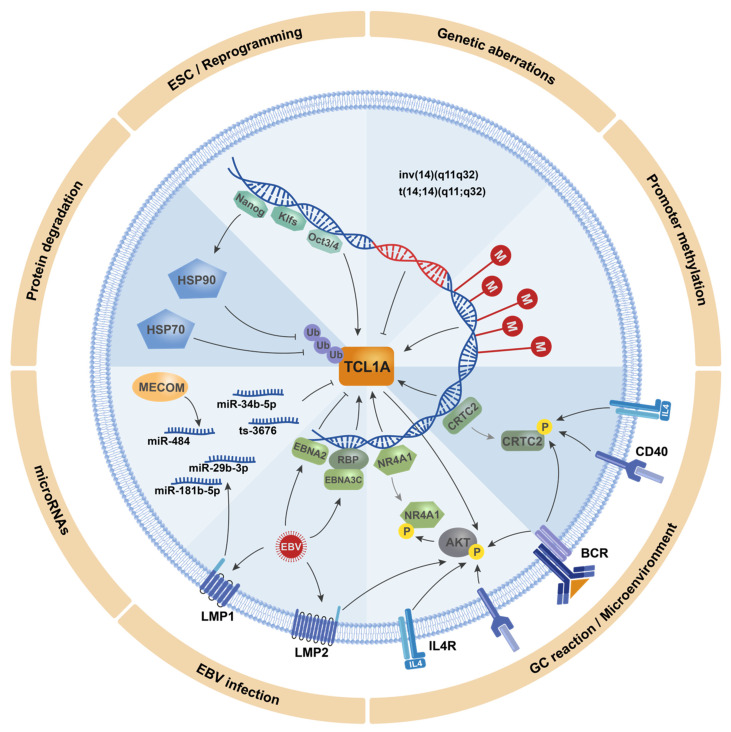
Schematic overview of the different modes of TCL1A regulation (clockwise categories). ESC/Reprogramming: In murine embryonic stem cells (ESCs) and induced pluripotent stem cells (iPSCs), *Tcl1a* expression is mediated by the transcription factors Nanog [75,76,81], Klf2/4/5 [60,82], and Oct3/4 [70]. Genetic aberrations: In T-cell prolymphocytic leukemia (T-PLL), an inversion or translocation of the *TCL1A* gene on chromosome 14 positions its locus under control by highly active regulatory regions of T-cell receptor genes. This prevents TCL1A’s post-thymic silencing and causes its constitutive expression [1]. Promoter methylation: During T-cell and B-cell development and maturation, a protracted epigenetic silencing of *TCL1A* expression is likely. B-cell lines have shown three different patterns of promoter methylation, that might reflect a successive increase in methylation along B-cell differentiation [83]. GC reaction/microenvironment: Signals via the BCR and/or via IL4R and CD40 ligation lead to phosphorylation and nuclear exclusion of CRTC2 [84] and NR4A1 [85], thereby repressing transcriptional activation of *TCL1A*. EBV infections: These signals by the microenvironment can also be mimicked by the Epstein–Barr virus (EBV) proteins LMP1 and LMP2, leading to repression of TCL1A [86,87]. Furthermore, the EBV protein EBNA2 represses [87], whereas EBNA3C increases, *TCL1A* expression [88]. MiRNAs: At the post-transcriptional level, TCL1A is regulated by several microRNAs (miRs), whose expressions are deregulated via co-deletion at 13q [89] and 17p [90], the EBV protein LMP1 [86], and the protein MECOM [29]. Protein degradation: Integrity of the TCL1A protein is regulated via the chaperones HSP70 [91] and HSP90 [76] that protect TCL1A from ubiquitination and subsequent degradation. Expression of *HSP90* is also regulated by the stem cell factor Nanog, which thereby mediates a bimodal regulation of TCL1A at the gene and protein level [76]. Grey arrows: dissociation from the *TCL1A* promoter.

The marked and safeguarding silencing of TCL1A during embryogenesis, as well as during T-cell and B-cell differentiation, is tightly regulated. As outlined, dysregulation of this machinery is associated with carcinogenesis. Understanding TCL1A’s transcriptional and translational regulation is, therefore, highly important. In the following, we present a comprehensive overview of modes of TCL1A (dys)regulation, including genomic aberrations, epigenetic modifications, dysregulation of TCL1A-targeting microRNAs (miRs), as well as modulations via altered signals by the microenvironment, which are likely mediated through some of these relays (Figure 2).

### 4.1. TCL1A Transcriptional Regulation in Embryonic Stem Cells and Cancer Stem Cells

Most of the evidence on TCL1A’s expression and transcriptional regulation in embryonic tissues derives from murine cells. *Tcl1a* was shown to be part of an embryonic expression signature important for self-renewal, also involving POU class 5 homeobox 1 (*Pou5f1*, encoding Oct3/4), Nanog homeobox (*Nanog*), SRY-box transcription factor 2 (*Sox2*), and the Myc proto-oncogene (*Myc*) [72,82]. In a global expression profiling of Oct3/4-manipulated mESCs, *Tcl1a* was identified as a direct transcriptional target of Oct3/4 by binding of the transcription factor (TF) to a sequence 410 bp upstream of the *Tcl1a* gene [70]. Furthermore, chromatin immunoprecipitation (ChIP) studies showed that the Kruppel-like factor (Klf) 2, 4, and 5 bind to the *Tcl1a* promoter region in mESCs [82], which could also be shown for Klf4 in iPSCs [60]. Knockdown of these factors led to a decrease in *Tcl1a* expression, suggesting their direct regulation of *Tcl1a* transcription [60,71,82]. The reactivation of this embryonic expression pattern also represents a plausible explanation for high TCL1A levels in solid tumors, especially in cancers harboring a cancer stem cell population [39,42,45]. Supporting this hypothesis, cancer stem cell-like cells, generated by cytotoxic T-lymphocyte-mediated immune selection of a cervical cancer cell line, showed a strong upregulation of TCL1A via transcriptional activation mediated by the stem-cell factor NANOG, which correlated with higher phosphorylation of AKT and increased tumorigenicity, as well as immune resistance [75].

### 4.2. TCL1A Promoter Activation

Next to stem-cell factors regulating *TCL1A* transcription, its 5′-promoter region contains a TATA box with cis-regulatory elements for several TFs expressed in somatic cells [85]. These include nuclear receptor subfamily 4 group A member 1 (NR4A1, also called Nur77) with its nerve growth factor-responsive element (NBRE), but also nuclear factor NF-κB, forkhead box protein O3 (FOXO3, also called FKHRL1), p53, and the TF SP1 [67].

SP1 mediates transactivation of the *TCL1A* core promoter by binding to three sites within its first 150 bp [92]. However, tissue-specific silencing of *TCL1A* expression does not seem to be dependent on mechanisms involving methylation of SP1 sites, as these were consistently un- or hypo-methylated in B-lymphoma cell lines of TCL1A-negative status. Furthermore, no differences in SP1 expression were seen in TCL1A-positive vs. negative cell lines [92]. Therefore, other mechanisms have to be involved in the protracted lymphocyte developmental stage-related decline in *TCL1A* gene expression [12,84].

One explanation could be a staged progression in CpG methylation of the *TCL1A* promoter. Overall, three discernable patterns of CpG DNA methylation in the promoter in *TCL1A*-silenced B-cell lines were noted, being methylations of only the CpGs in 5′-flanking regions, methylations of CpGs in 5′- and 3′-regions, and methylations spanning the whole promoter. Treatment with an inhibitor of DNA methylation, 5-azacytidine, was able to restore the expression of *TCL1A* in these cell lines, arguing for epigenetic regulation of *TCL1A* promoter repression [83].

There are also repressive relationships of TFs predicted to bind to *TCL1A*’s transcriptional start site, but most of them are implicated by circumstantial evidence from associative data. Examples are FOXO3 and p53 [67,93]. A negative feedback loop of the TCL1A-AKT axis has been identified as well. Here, NR4A1 is activated by phosphorylated AKT and prevented from binding to the NBRE of the *TCL1A* promoter, resulting in repression of *TCL1A* transcription [85]. This implicates that under normal conditions, lymphocyte activation via TCL1A-mediated auto-phosphorylation of AKT dimers entails subsequent safeguarding repression of this proto-oncogene. This autoregulation might be disturbed in lymphomatous T cells or B cells.

Next to this negative feedback loop, there is additional B-cell activation-induced repression of *TCL1A*, with a safeguarding significance during the GC reaction of B cells [84]. In fact, experimentally sustained TCL1A expression during the GC reaction is oncogenic [94]. A CREB response element in the *TCL1A* promoter was identified and its activation was independent of the phosphorylation of CREB but depended on the CREB-regulated transcription coactivator 2 (CRTC2, also called TORC2). Interestingly, GC-associated stimulation via CD40 ligand (CD40L)/interleukin 4 (IL4) or via BCR engagement resulted in phosphorylation of CRTC2, leading to its nuclear exclusion and subsequent partial *TCL1A* repression, while other pCREB/E1A binding protein p300 (EP300)-dependent genes were activated via CREB phosphorylation and EP300 recruitment. However, a reduction in TCL1A levels of only 40% at more than 95% CRTC2 repression implicates that the control of the *TCL1A* gene in the GC B-cell involves other regulatory levels as well [84].

Next to lymphocytes, TCL1A is also highly expressed in pDCs and in the derived BPDCN [32]. The TF TCF4 strongly expressed in pDCs and crucial for their lineage commitment and maintenance, was shown to bind to the *TCL1A* promoter via ChIP-seq analyses [95,96]. Knockdown of TCF4 reduced the expression of *TCL1A*, suggesting a positive regulation by this TF [96]. Furthermore, a negative correlation of *TCL1A* and expression of the ETS Variant TF 6 (*ETV6*) in BPDCN and in B-cell acute lymphoblastic leukemia (B-ALL) implicates an additional mode of *TCL1A* transcriptional regulation [97].

Notably, there is also evidence for the role of single nucleotide polymorphisms (SNPs) in the regulation of *TCL1A*. A genome-wide association study in women treated with aromatase inhibitors (AIs) for early breast cancer identified the SNP rs11849538 close to the 3′ end of *TCL1A* that generates an estrogen response element. In cell lines carrying this SNP, an estrogen-dependent expression of *TCL1A* was suggested [98,99]. This SNP was associated with a higher risk for the development of musculoskeletal pain under AI treatment [98]; however, this finding could not be validated in an independent cohort [100].

### 4.3. Posttranscriptional Regulation of TCL1A by Micro RNAs

Evidence on the relevance of the contribution of miRs to TCL1A suppression derives from a tg mouse model of full-length *TCL1A* with its preserved 3′ and 5′ untranslated regions (UTRs) [101]. In contrast to the initial *Eμ-TCL1A* model, where only the human *TCL1A* open reading frame was overexpressed [16], this model allows for the inhibitory impact by TCL1A-regulating miRs. Fittingly, the phenotype of the induced CLL is milder than in the classical *Eμ*-*TCL1A* tg mouse [101].

Several miRs were identified to negatively regulate TCL1A at the posttranscriptional level. MiR-29b-3p and miR-181b-5p, shown to repress TCL1A, inversely correlated in their expression with TCL1A levels across CLL subsets defined by features of clinical aggressiveness [102]. Genomic losses of such negative regulators as miRs might be one mechanism causing the increased TCL1A levels in human CLL besides histogenetic determination and transcriptional influences [12,28,103]. In support, particularly high TCL1A levels are observed in the aggressive subsets of CLL that are characterized by chromosomal losses at 11q22 (*ATM*) [28] and 17p (*TP53*) [29], with a suggested codeletion of TCL1A repressive miR-34b-5p [90] and tRNA-derived small RNA (tsRNA)-3676 (before known as miR-3676) [89], respectively. Next to deletions, some loss-of-function mutations of ts-3676 were also identified in around one percent of CLL patients [89].

In addition, we identified miR-484 to target the 3′-UTR of TCL1A in CLL [29]. MiR-484 showed a transcriptional downregulation in a large cohort of CLL patients, mediated via a downregulation of the TF MDS1 and EVI1 complex locus (MECOM, also called EVI1). Accordingly, we observed an inverse correlation of *MECOM* and *TCL1A* expression in a large cohort of CLL. *TCL1A* and *MECOM* showed a strong interactive clinical hazard prediction in prospectively treated patients, suggesting a contribution of the described regulatory circuit to an aggressive cellular and clinical phenotype in CLL [29].

### 4.4. Posttranslational Regulation of TCL1A

Evidence on the regulation by posttranslational modifications of the TCL1A protein is sparse. However, one site in TCL1A has been identified that, when phosphorylated potentially by the glycogen synthase kinase-3β (GSK3β), decreases the interaction of TCL1A with its client protein hnRNPK [44]. Furthermore, there is recent evidence for a regulation of TCL1A protein integrity by chaperones. The heat shock 70 kDa protein 1A (HSPA1A, in the following HSP70) was shown to bind to TCL1A and to protect it from ubiquitination and subsequent degradation. Accordingly, inhibition of HSP70 led to a reduction in TCL1A protein in primary CLL cells and impaired signaling of the NF-κB cascade [91]. Given that HSP70 is overexpressed in CLL cells, this could represent a potential mode of dysregulation of this proto-oncogene [104]. A similar regulation was identified in an immune-edited tumor cell line with a stem cell-like phenotype. Here, heat shock protein 90 alpha family class A member 1 (HSP90AA1, in the following HSP90) was identified as a TCL1A-stabilizing chaperone by counteracting its ubiquitination and degradation and thereby reinforcing the TCL1A-AKT axis [76]. The transcriptional regulator of *TCL1A*, NANOG, was also identified to induce transcription of *HSP90*, thereby mediating a bimodal regulation of TCL1A at the gene and protein level [76].

## 5. Exogeneous Triggers of TCL1A-Regulating Mechanisms

### 5.1. Regulation of TCL1A Levels by the Microenvironment

What triggers these molecular modes of TCL1A (dys)regulation as histogenetically driven (incl. differentiation-associated) programs? Several publications suggest the role of micromilieu-derived stimuli in regulating TCL1A levels in B-cell tumors. In sections of CLL and other B-cell tumors in lymph nodes, spleens, and bone marrow, and as mimicked in stimulated suspension cultures, strong TCL1A expression in the resting cells was paralleled by near-complete losses of TCL1A protein in the fraction of Ki67^+^ proliferating paraimmunoblasts [12,28]. This pattern was best characterized as cell-cycle-related oscillating levels of TCL1A, likely regulated at the level of protein turnover. This unexpectedly dynamic pattern at the single-cell level was particularly prominent in the pseudofollicular proliferation centers of CLL that are enriched for bystander T cells. In subsequent in vitro studies, cytokines typically secreted from such supportive T cells, mainly CD40L and IL4, induced proliferation and differentiation and ultimately reduced the overall TCL1A expression in these long-term CLL suspension cultures [28]. This latter phenomenon might be mediated by the repression of transcriptional activation via NR4A1 and CRCT2 or a reduced TCL1A protein integrity [84,85]. Based on this, we postulate the existence of fast-acting cell-cycle-dependent ways to eliminate a mainly anti-apoptotic protein when entry from the G0/G1 arrest into a proliferative phase is necessary, which is paralleled by differentiation-associated transcriptional programs of TCL1A silencing.

As another source of TCL1A-regulating milieu-derived stimuli, direct cell–cell contact of leukemia with bone marrow stromal cells (BMSCs) was identified [105]. In contrast to the suppressive impact of T-cell-derived stimuli [28], BMSC contact led to the upregulation of TCL1A mRNA and protein. Gene expression profiling revealed that *TCL1A* was among the top genes upregulated in CLL cells by cocultures on BMSC. Stroma-mediated increases in TCL1A were also associated with decreased levels of TCL1A-repressive miRs (miR-29b, miR-181b, miR-34b, and miR-484) [105]. These findings demonstrate that the microenvironment has a proactive role in the regulation of TCL1A in B-cell tumors, i.e., CLL, and that a fine-tuning modulation via miRs is involved therein as well. This provides a further molecular rationale for targeting the lymphoma-milieu crosstalk.

### 5.2. Alterations of TCL1A Expression in EBV Infection

The regulation of TCL1A seems perturbed also in the context of B-cellular Epstein–Barr virus (EBV) infection. The majority of EBV-infected B-cell non-Hodgkin lymphomas (B-NHL) appear to be positive for TCL1A to a higher degree than their EBV-negative counterparts [106,107]. In support of positive regulation of TCL1A expression via EBV, infection of BL cell lines and a MM cell line by EBV induced upregulation of their TCL1A expression [106,108]. Furthermore, lymphoblastoid cell lines (LCLs), generated from B cells infected with EBV in vitro, showed an upregulation of TCL1A that was dependent on the interaction of the EBV nuclear antigen 3C (EBNA3C) with the host recombination signal binding protein for immunoglobulin kappa J region (RBPJ) [88].

However, there is also contradicting data for a repressive influence of EBV on TCL1A expression. EBV infection of DLBCL cell lines in vitro reduced TCL1A levels in an EBNA2-dependent manner [87]. Furthermore, overexpression of the EBV-derived latent membrane protein 1 (LMP1) was shown to reduce expression of TCL1A in several B-cell lines [86,87], in part mediated by the overexpression of the TCL1A-targeting miR-29b [86]. As LMP1 and LMP2 mimic constitutive activation of CD40 and the BCR, respectively, they might activate the TCL1A-repressive signals mediated by these cascades (described in Section 4.2 and Section 5.1) as well [109,110,111].

Several possible reasons might, at least in part, explain these contradicting results, besides the general limitations of artificial EBV introduction into cell lines. First, the expression of certain EBV gene products is restricted to a specific latency pattern of EBV infection, which, in turn, is strongly impacted by the host immune competence. Cells with a latency I profile only express *EBNA1*, besides some non-coding genes. In contrast, proteins negatively regulating TCL1A expression are expressed in latency II and III profiles. Accordingly, most cell lines that showed an upregulation of TCL1A after infection in vitro expressed a type I latency. Second, the cell of origin might determine to which extend the expression of TCL1A can be manipulated by EBV infection. As discussed above, epigenetic methylation of the *TCL1A* promoter can be a mechanism by which the *TCL1A* gene is silenced during B-cell differentiation. EBV infection may be unable to counteract a strong epigenetic silencing in some cell types. This could explain why post-GC derived, TCL1A-negative primary effusion lymphomas (PEL) did not upregulate TCL1A upon EBV infection [112], whereas expression of TCL1A in BL and DLBCL lines was modulated by EBV [86,87,88,106]. However, post-GC-derived AIDS-DLBCL expresses TCL1A at a frequency equivalent to naïve/GC-derived B-cell lymphomas in immune-competent individuals, although often expressing type II/III latency.

From the data on the impact of EBV on TCL1A levels, we postulate that generally, TCL1A expression is also influenced by severe immune dysfunction. In alignment with the findings of a TCL1A-repressive impact by T-cell-derived CD40L/IL4 stimuli or by BCR signals (see Section 4.2 and Section 5.1) [12,28,84], a depleted T-cell compartment or severe EBV infection might antagonize the “B-cell developmental” (see Section 2.2) TCL1A downregulation. Although some of the discrepancies can be explained by the above points, they do not fully resolve the heterogeneity of TCL1A expression among the various B-cell lymphoma subtypes in the context of EBV [112,113].

## 6. Discussion

The adaptor protein TCL1A has vital functions in reproduction, development, and adaptive immunity. Identification of its overexpression and oncogenic role in lymphatic and in part in other tumors, particularly in T-PLL, CLL, and BPDCN, has established its diagnostic and prognostic marker properties [55,114]. In non-neoplastic settings, its expression was utilized as a distinct prediction marker, as high TCL1A expression in peripheral blood mononuclear cells of patients undergone kidney transplantation correlated with tolerance after transplantation, which mainly results from a higher naïve B-cell population in tolerant patients (reviewed in [115]).

This review provides a comprehensive overview of the different modes of (dys)regulation of this prototypic member of the TCL1 oncogene family. Tight regulation of TCL1A’s timely silencing is important, given the multiple oncogenic functions of TCL1A in mature lymphocytes and likely in other cell lineages as well. Mechanistic models of pathogenic TCL1A upregulation were first restricted to genomic translocations that involve its gene locus, as shown in T-PLL. However, to date, multi-faceted ways of TCL1A (dys)regulation have been identified, mostly by data from B-cell malignancies. In addition to promoter hypomethylation of the *TCL1A* gene, dysregulation of TCL1A-targeting miRs, but also chaperone-mediated protection from protein degradation were identified. Additionally, potential phosphorylation sites have been identified in murine and human TCL1A; however, their role in the function and regulation of the protein is still uncertain [44,116].

Exogeneous triggers of such TCL1A (dys)regulation in malignant B cells originate from the microenvironment. TCL1A suppressive signals from T-cell-derived (humoral) factors or upregulation of TCL1A in CLL cells in the bone marrow niche via cell–cell contacts with BMSC represent relevant influxes. However, the (disturbed) homeostasis between these suppressive vs. activating impacts on TCL1A levels is still inadequately addressed. A better characterization of these specific sources of TCL1A regulation and their molecular executions within a comprehensive regulatory network of TCL1A could be of benefit considering the increasing application of inhibitors disrupting the crosstalk of lymphatic tumor cells with their microenvironment.

In solid tumors, overexpression of TCL1A might originate from the reactivation of an embryonic program, including expression of *NANOG*, *OCT3/4*, *MYC*, etc., during epithelial cell transformation. Although there is some evidence for a prognostic impact of TCL1A expression [30], its functional relevance for oncogenic signaling in solid carcinogenesis should be further defined.

For several lymphatic tumors, TCL1A’s transforming capacity is firmly established by the highly recurrent genomic *TCL1A* rearrangements in T-PLL [25,46] and by *TCL1A*-tg mouse models [13,16]. However, it remains unclear whether these leukemias/lymphomas are centrally (co) initiated by TCL1A (likely the case in T-PLL) and whether they retain a TCL1A dependence. This should be addressed in models of genetic depletion of TCL1A in murine and human T-cell and B-lymphocytic leukemias.

As high TCL1A expression correlates with an inferior clinical outcome, an effect of TCL1A on tumor sustenance is likely, and its impact on several different oncogenic pathways makes it an appealing target. However, as a small adaptor molecule without catalytic domains, its pharmaceutical targeting is very challenging. New avenues in such specific therapeutic interventions would include the profiling for TCL1A dimerization inhibitors or non-peptide chemotypes that intercept in TCL1A-complex formation. It is also highly intriguing that TCL1A peptide sequences (TCL1A71-78 LLPIMWQL) were identified as an HLA-A*0201 binding T-cell epitope [117]. TCL1A71-78 peptide-specific T cells were shown to be present in CLL patients and to lyse autologous tumor cells but not normal B cells in vitro in an HLA-A2-restricted manner [117]. This suggests that TCL1A is processed and presented on the surface of CLL cells for recognition by cytotoxic T cells and that it can serve as a novel target for vaccinations or in other immune-therapeutic strategies such as TCL1A-targeting CAR T cells [118].

## 7. Conclusions

In this review, we summarized the current knowledge on the oncogenic function and transcriptional and translational regulation of the TCL1A proto-oncogene, resulting in a mechanistic concept of its context-dependent transforming capacities and multiple modes of (dys)regulation (Figure 2). This improved biological understanding forms the basis for future work on approaches to interfere in tumorigenic processes mediated by TCL1A or to target this molecule directly, i.e., in TCL1A-overexpressing leukemias.

## Figures and Tables

**Table 1:** Expression of TCL1A in different malignancies.

Entity	N	TCL1A Expression [% Cases]	Reference	Prognostic Implications
Leukemia/Lymphoma				
T-cell leukemias/lymphomas				
T-cell prolymphocytic leukemia	38–59	71–75	[32,33,46]	Shorter OS [26]
T-(acute) lymphoblastic leukemia/lymphoma	7–47	14–36	[32,33]	-
Adult T-cell leukemia/lymphoma	5	20	[32]	-
B-cell leukemias/lymphomas				
B-(acute) lymphoblastic leukemia/lymphoma	4–55	75–85	[12,47]	-
Mantle cell lymphoma	5–58	84–100	[12,47,48]	Shorter LSS [38]
Burkitt Lymphoma	5–16	94–100	[12,47,49]	-
Follicular lymphoma	11–49	57–75	[12,47,48]	-
Diffuse large B-cell lymphoma	11–15	18–60	[12,47,48,49]	Shorter OS [50]
Pediatric diffuse large B-cell lymphoma	16	31	[51]	-
CD5- Lymphoproliferative disorder	2	100	[12]	-
MALT lymphoma	9–23	11–83	[12,48]	-
Lymphoplasmacytic lymphoma	4	75	[48]	-
Small lymphocytic lymphoma	2	100	[48]	-
Chronic lymphocytic leukemia	11–126	90–100	[28,47]	Shorter TFS/PFS [24,38]
Cutaneous B-cell lymphoma	9–25	20–55	[47,52]	-
Waldenström macroglobulinemia	57	79	[53]	No [53]
Myeloid neoplasms				
Extramedullary myeloid cell tumors	14	7	[32]	-
Blastic plasmacytoid dendritic cell neoplasm	12–91	83–99	[32,35]	-
Solid tumors				
Bladder cancer	10	40	[42]	-
Prostate cancer	5	80	[42]	-
Colon cancer	5	60	[42]	-
Colorectal cancer	278	>70	[30]	Shorter DSS [30]
Hepatocellular carcinoma	65	>44	[44]	Shorter OS [44]
Germ cell tumors				
Classical seminoma	13–55	77–100	[9,39,40]	-
Embryonal carcinoma	34	9	[40]	-
Intratubular germ cell neoplasia	40–50	100	[39,40]	-
Spermatocytic seminoma	6	17	[40]	-
Ovarian dysgerminoma	25	100	[54]	-
Ovarian yolk sac tumor	29	59	[54]	-

N = cohort size, range from lowest to highest in the different studies; LSS = leukemia-specific survival; OS = overall survival; TFS = treatment-free survival; PFS = progression-free survival; DSS = disease-free survival.
